# Identification of Key CircRNAs Related to Pulmonary Tuberculosis Based on Bioinformatics Analysis

**DOI:** 10.1155/2022/1717784

**Published:** 2022-04-04

**Authors:** Qin Yuan, Zilu Wen, Ke Yang, Shulin Zhang, Ning Zhang, Yanzheng Song, Fuxue Chen

**Affiliations:** ^1^School of Life Sciences, Shanghai University, Shanghai, China; ^2^Department of Scientific Research, Shanghai Public Health Clinical Center, Fudan University, Shanghai, China; ^3^Department of Immunology and Microbiology, Shanghai Jiao Tong University School of Medicine, Shanghai, China; ^4^Tuberculosis Research Center, Shanghai Public Health Clinical Center, Shanghai, China; ^5^Department of Hepatic Surgery, Fudan University Shanghai Cancer Center, Shanghai, China; ^6^Department of Thoracic Surgery, Shanghai Public Health Clinical Center, Fudan University, Shanghai, China

## Abstract

Pulmonary tuberculosis (TB) is a chronic infectious disease that is caused by respiratory infections, principally *Mycobacterium tuberculosis*. Increasingly, studies have shown that circular (circ)RNAs play regulatory roles in different diseases through different mechanisms. However, their roles and potential regulatory mechanisms in pulmonary TB remain unclear. In this study, we analyzed circRNA sequencing data from adjacent normal and diseased tissues from pulmonary TB patients and analyzed the differentially expressed genes. We then constructed machine learning models and used single-factor analysis to identify hub circRNAs. We downloaded the pulmonary TB micro (mi)RNA (GSE29190) and mRNA (GSE83456) gene expression datasets from the Gene Expression Omnibus database and performed differential expression analysis to determine the differentially expressed miRNAs and mRNAs. We also constructed a circRNA–miRNA–mRNA interaction network using Cytoscape. Gene Ontology enrichment analysis and Kyoto Encyclopedia of Genes and Genomes pathway analysis were used to predict the biological functions of the identified RNAs and determine hub genes. Then, the STRING database and cytoHubba were used to construct protein-protein interaction networks. The results showed 125 differentially expressed circRNAs in the adjacent normal and diseased tissues of pulmonary TB patients. Among them, we identified three hub genes associated with the development of pulmonary TB: hsa_circ_0007919 (upregulated), hsa_circ_0002419 (downregulated), and hsa_circ_0005521 (downregulated). Through further screening, we determined 16 mRNAs of potential downstream genes for hsa-miR-409-5p and hsa_circ_0005521 and established an interaction network. This network may have important roles in the occurrence and development of pulmonary TB. We constructed a model with 100% prediction accuracy by machine learning and single-factor analysis. We constructed a protein-protein interaction network among the top 50 hub mRNAs, with FBXW7 scoring the highest and SOCS3 the second highest. These results may provide a new reference for the identification of candidate markers for the early diagnosis and treatment of pulmonary TB.

## 1. Introduction

Pulmonary tuberculosis (TB) is a contagious disease that is caused by the slow-growing *Mycobacterium tuberculosis* (MTB), which is spread by aerosols [1]. The primary pathological feature of pulmonary TB is necrotizing granuloma infiltration [2]. TB infections mainly occur in the lungs but may also cause problems in other parts of the body [3]. Pulmonary TB has a long incubation period. When MTB enters the lungs, it usually forms capsules (granulomas), which are harmless in the lungs and cause latent pulmonary TB infection [4–6]. However, these capsules may be activated in the future and develop into active pulmonary TB [7]. Although pulmonary TB can be cured, it remains one of the ten leading causes of death worldwide, particularly in developing countries [8, 9]. Latent pulmonary TB infections are asymptomatic, making this difficult to detect and treat on time [10]. Therefore, there is great interest in finding effective biomarkers for the early diagnosis and/or treatment of latent pulmonary TB [11].

Circular (circ)RNAs are a type of noncoding RNA molecule that lack a 5′ cap and 3′ tail and form a single-stranded circular structure by covalent bonds [12]. CircRNAs are formed by reverse splicing and are mainly divided into all-exon circRNAs, circRNAs composed of introns and exons, and lasso-shaped circRNAs composed of introns [13, 14]. CircRNAs are abundant and present in bodily fluids such as blood, saliva, cerebrospinal fluid, and urine [15]. Compared with linear RNAs, circRNAs are more stable and more resistant to degradation by RNase R. Therefore, circRNAs can be selectively enriched during sample processing [16, 17]. Compared with other types of RNAs, circRNAs are more suitable as candidate molecules for molecular diagnostic biomarkers and could be effective therapeutic targets [18].

Additionally, circRNAs can be used as micro (mi)RNA sponges to bind miRNAs through miRNA response elements, releasing the degradation or translational inhibition of downstream target mRNAs by disrupting the competitive endogenous (ce)RNA mechanism [19]. CircRNAs bind to and regulate the functions of RNA-binding protein (RBP), changing the stability of mRNAs. CircRNAs can also directly encode proteins [20–22]. According to a report by Li et al., who analyzed the expression profile of circRNAs in nonsmall cell lung cancer, a circRNA named CircNDUFB2 could inhibit nonsmall cell lung cancer progression by decreasing the stability of insulin-like growth factor 2 mRNA-binding proteins (IGF2BPs) and activating antitumor immunity [23]. Wang et al. studied the expression profile of circRNAs in the lung tissue of mice infected with influenza A virus (IAV) using high-throughput sequencing and RNA sequencing. They identified many differentially expressed circRNAs between the IAV group and the control group. Bioinformatic analysis revealed a potential role of circRNAs in the IAV-induced lung injury model [24]. Zhou et al. conducted RNA sequencing and correlation analysis on liver biopsies of untreated patients with chronic hepatitis B and healthy controls. They found that there was a strong positive correlation between hsa_circ_0000650 and TGFB2 expression and a negative correlation between hsa_circ_0000650 and miR-6873-3p expression. They confirmed that there were different circRNAs and circRNA/miRNA interactions in patients with chronic hepatitis B [25].

To summarize, many studies have shown that circRNAs are associated with the occurrence and development of a variety of respiratory and infectious diseases [3, 26–28]. Currently, there are few studies on circular RNAs and pulmonary TB. Pulmonary TB-related circRNA research could be important for the early diagnosis and treatment of pulmonary TB [29]. In this study, we sought to identify differentially expressed circRNAs and their downstream mRNAs using tissues from pulmonary TB patients to identify hub genes involved in pulmonary TB infection and provide references for the early diagnosis and treatment of pulmonary TB.

## 2. Materials and Methods

### 2.1. Data Collection

The Shanghai (Fudan University) Public Health Clinical Center provided all patient data used in this study. They performed positron emission tomography (PET) scans on the lungs of nine pulmonary TB patients and whole transcriptome sequencing of lung tissues with high PET (high metabolic activity) and normal PET (low metabolic activity) uptake. For raw sequencing data, please refer to PRJNA795290 (4∗2 samples) from the Sequence Read Archive (SRA) database and GSE158767 (5∗2 samples) from the Gene Expression Omnibus (GEO) database. All patients signed an informed consent form before obtaining tissue samples. RNA extraction, library construction, and sequencing were performed in the same way as previously described [30]. In this paper, we analyzed 8286 differentially expressed genes (DEGs) by bioinformatics.

### 2.2. Differential Expression Analysis of CircRNAs

First, the raw data were corrected, filtered, and normalized using the R package. Analyses of differentially expressed RNAs included fold change (FC), *P* value, and false discovery rate (FDR). The criteria for selecting differentially expressed circRNAs were ∣log2*FC* | >1 and *P* value < 0.05. Scatter plots, volcano plots, and heat maps were used to show the differently expressed circRNAs.

### 2.3. Feature Selection

In machine learning applications, the number of features is often large, and there may be irrelevant features. With more features, the resulting model will be more complex. Feature selection can eliminate irrelevant or redundant features, reduce the number of features, improve model accuracy, reduce the running time, and simplify the model. We identified hub circRNAs using eight feature screening methods: CfsSubsetEval-BestFirst, PrincipalComponents-Ranker-T, CorrelationAttributeEval-Ranker-T, GainRatioAttributeEval-Ranker-T, InfoGainAttributeEval-Ranker-T, OneRAttributeEval-Ranker-T, ReliefFAttributeEval-Ranker-T, and SymmetricalUncertAttributeEval-Ranker-T.

### 2.4. Building the Machine Learning Models

We used 13 algorithms to build the models: ZeroR, Logistic, SMO, IBK, AttributeSelectedClassifier, OneR, DecisionStump, HoeffdingTree, J48, LMT, RandomForest, RandomTree, and REPTree. Comparisons of the average accuracy of the machine learning models established by different feature selection methods were then conducted. We also performed statistical and univariate analyses of the number of occurrences of circRNAs in various models to identify hub circRNAs.

### 2.5. Screening the Downstream miRNAs and mRNAs of CircRNAs and Constructing the CircRNA–miRNA–mRNA Regulatory Network

Predictions of potential miRNAs targets of the circRNAs were made through the ENCORI website (http://starbase.sysu.edu.cn/). We screened the pulmonary TB miRNA gene chip (GSE29190) dataset from the Gene Expression Omnibus (GEO) database (http://www.ncbi.nlm.nih.gov/geo) for differentially expressed miRNAs. The criteria for selecting differentially expressed miRNAs were ∣log2*FC* | >1 and *P* value < 0.05. Predictions of potential mRNAs downstream of miRNAs were made by the miRTarBase database (http://mirtarbase.mbc.nctu.edu.tw/php/index.php). Then, the GEO database of pulmonary TB mRNA gene chip (GSE83456) was used to screen for differentially expressed mRNAs. The criteria for selecting differentially expressed mRNAs were |log2*FC* | >1 and *P* value < 0.05. Finally, Cytoscape (http://www.cytoscape.org/) was used to construct the circRNA–miRNA–mRNA interaction network.

### 2.6. Biological Function Analysis

Gene ontology (GO) enrichment analysis and Kyoto Encyclopedia of Genes and Genomes (KEGG) pathway analysis were performed using the DAVID database (https://david.ncifcrf.gov) for the differentially expressed mRNAs satisfying ∣log2*FC* | >1 and *P* value < 0.05. GO analysis is divided into three categories, cellular component (CC), molecular function (MF), and biological process (BP), and summarizes the biological functions, pathways, or cellular localization of gene enrichment. We used the KEGG database for pathway enrichment analysis.

### 2.7. Construction of Protein-Protein Interaction (PPI) Networks

For the 372 mRNAs, we identified from the previous screen; we analyzed them using the STRING database (https://string-db.org/) and uncovered information on the interactions of these proteins. Each node in the PPI network represents a protein. To identify the key nodes in the PPI network, we calculated the top 50 hub genes using the cytoHubba function in Cytoscape (http://www.cytoscape.org/). We then constructed a PPI network on the basis of these 50 hub genes.

## 3. Results

### 3.1. Preliminary Screening of Differentially Expressed Genes in Pulmonary TB

**Figure 1:**
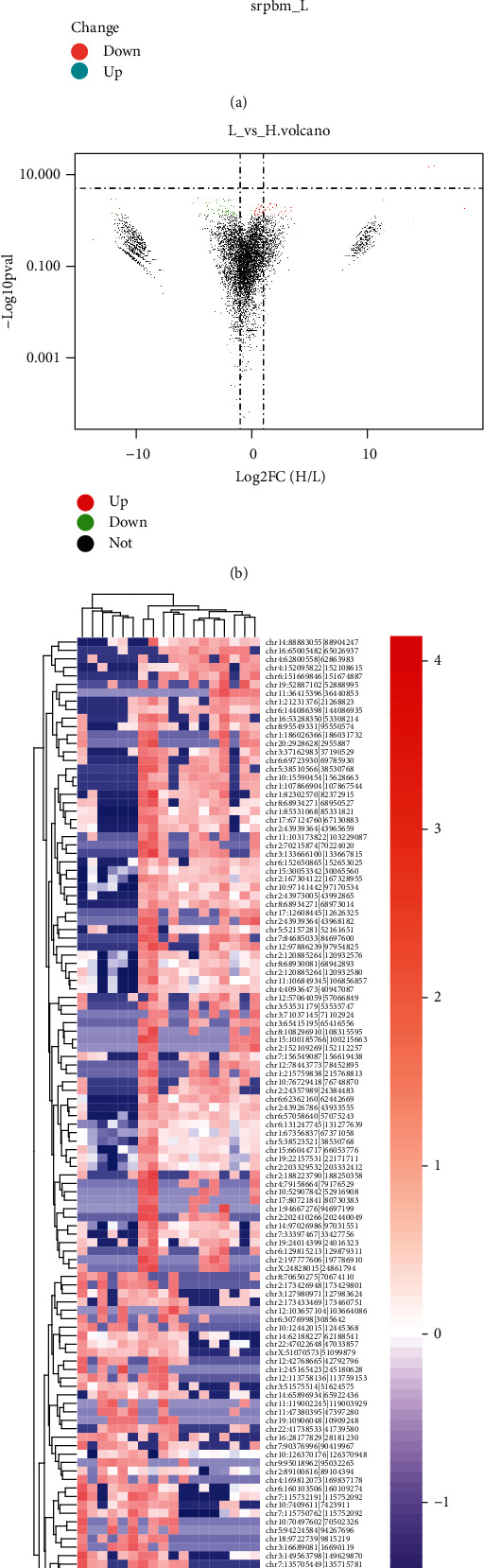
Sequencing data and differential expression analysis. (a) Scatter plot analysis of circRNA expression; (b) volcano plot of differential circRNA expression; (c) heat map of cluster analysis of the differentially expressed circRNAs.

We initially conducted a differential expression analysis of pulmonary TB patient sequencing data. The larger the difference in DEGs between normal and diseased tissues, the stronger the association between the disease state and the DEG. A scatter plot was used to show gene expression. The genes clustered towards the middle show less differential expression, while those dispersed towards the sides show larger differential expression. Points on both sides are more likely to be disease hub genes (Figure 1(a)). The basic conditions for screening DEGs were a statistical significance measure *P* < 0.05 and the absolute change in differential gene expression (fold change, FC) > 2. There were 125 circRNAs expressed in the adjacent normal and diseased tissues of pulmonary TB patients that satisfied *P* value < 0.05 and ∣log2*FC* | >1, among which 50 were upregulated and 75 were downregulated. There were more downregulated genes in normal tissues compared with diseased tissues (Figure 1(b)). Among the differentially expressed circRNAs, the top 10 up- and downregulated circRNAs are listed in Table 1. Next, we drew a heat map for cluster analysis on basis of circRNA expression in the different samples (Figure 1(c)). The results showed that there were more upregulated genes in diseased tissues. These conclusions were consistent with the volcano plots.

### 3.2. Building the Machine Learning Models

#### 3.2.1. Construction of the Whole Gene Prediction Models

These machine learning models were built from the 125 differently expressed circRNAs. The prediction accuracy of the Logistic, SMO, and IBK algorithms were the highest, reaching 100%; the accuracy of the J48 algorithm also reached 89% (Table 2).

#### 3.2.2. Feature Selection

The pathogenesis of pulmonary TB is complex. Predictive modeling of pulmonary TB hub genes by machine learning was then performed with the 125 differentially expressed circRNAs. First, we performed feature selection to screen the major influencing factors among the 125 circRNAs; the hub circRNAs were determined by eight feature selection methods. We constructed a machine learning model on the basis of the 125 circRNAs and feature-screened circRNAs. We built models separately using 13 different algorithms: ZeroR, Logistic, SMO, IBK, AttributeSelectedClassifier, OneR, DecisionStump, HoeffdingTree, J48, LMT, RandomForest, RandomTree, and REPTree (Tables S1–S9).

**Figure 2:**
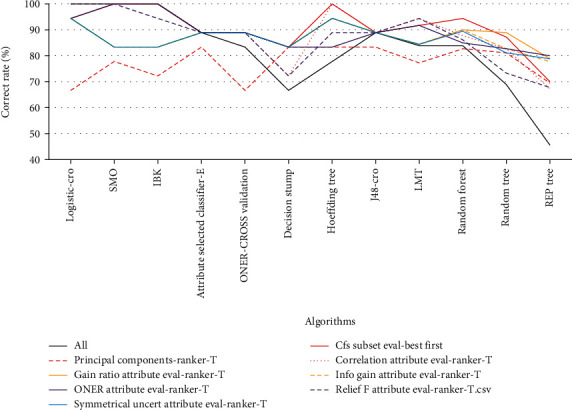
Comparison of the average accuracy of machine learning models built by different feature screening methods.

CorrelationAttributeEval-Ranker-T feature screening had four algorithms with a 100% accuracy rate (Logistic, SMO, IBK, and HoeffdingTree). The CfsSubsetEval-BestFirst feature screening method was the next best, and its three algorithms (SMO, IBK, and HoeffdingTree) had a 100% accuracy rate. OneRAttributeEval-Ranker-T feature screening had two algorithms (SMO and IBK) that showed a 100% accuracy rate. CfsSubsetEval-BestFirst feature screening also had two algorithms with a 100% correct rate (Logistic and SMO). Only CorrelationAttributeEval-Ranker-T had a better accuracy rate than the model constructed with 125 circRNAs. Therefore, the CorrelationAttributeEval-Ranker-T feature screening method was more suitable for the 125 circRNAs (Table 2 and Figure 2). Among the eight feature screening methods, four reached 100% model correctness. These four methods contained 29 circRNAs, counting their occurrences. There were 14 circRNAs that appeared more than once in these four methods. Among them, hsa_circ_0007919, chr10:15590454|15628663, and hsa_circ_0002419 appeared in all four methods. This suggested that these genes may be closely related to the occurrence and development of pulmonary TB (Table 3).

### 3.3. Univariate Analysis and Confirmation of the Key CircRNAs

#### 3.3.1. Univariate Analysis

To further clarify the effect of circRNAs on pulmonary TB, we built machine learning models for 14 circRNAs and performed univariate analysis. We calculated the average of the models with correct rates of over 80%, with random seeds taken from 1 to 10 (Table 4). The results showed that each circRNA had at least one algorithm with an accuracy greater than 80%. Among them, hsa_circ_0002419 had a strong correlation with pulmonary TB, and the accuracy of the four algorithms was 94%. Hsa_circ_0005521 had a strong correlation, and the correct rate was 89%.

#### 3.3.2. Confirmation of the Key CircRNAs

**Figure 3:**
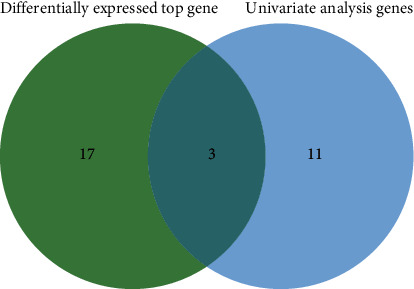
Cross-analysis of the top differentially expressed genes and genes identified by univariate analysis.

To identify hub genes in the development of pulmonary TB, we made Venn diagrams that included 20 DEGs and 14 univariate analysis genes using Jvenn. We uncovered three hub circRNAs: hsa_circ_0007919 (upregulated), hsa_circ_0002419 (downregulated), and hsa_circ_0005521 (downregulated). We identified these three circRNAs as hub genes in the development of pulmonary TB (Figure 3 and Table 1).

### 3.4. Downstream Gene Prediction and Biological Function Analysis

#### 3.4.1. The Downstream Genes of the Identified CircRNAs

**Figure 4:**
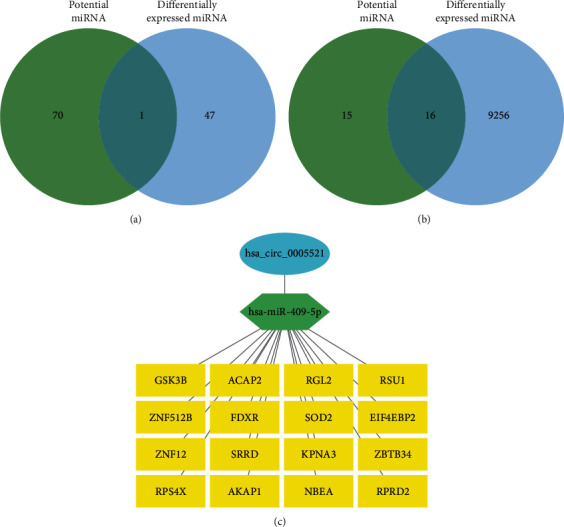
Analytical screening of downstream miRNAs and mRNAs. (a) Screening of downstream miRNAs using Jvenn cross-analysis of potential miRNAs and the differentially expressed miRNAs. (b) Screening of downstream mRNAs using Jvenn cross-analysis of potential mRNAs and the differentially expressed mRNAs. (c) The circRNA–miRNA–mRNA interaction network.

The most interesting function of circRNAs is to serve as molecular sponges for miRNAs by binding and influencing miRNA expression. The potential miRNAs that interact with the hub circRNAs were predicted using the ENCORI database. The downstream mRNAs of the miRNAs were predicted by the miRTarBase database (Table 5). We used the miRNA Gene Chip GSE29190 of pulmonary TB from the GEO database and screened out 47 differentially expressed miRNAs (*P* < 0.05). We screened out potential miRNAs and differentially expressed miRNAs by Jvenn, which identified hsa-miR-409-5p (Figure 4(a)). The upstream molecule of hsa-miR-409-5p is hsa_circ_0005521, and the total number of potential downstream mRNAs is 31.

We used the mRNA Gene Chip GSE83456 of pulmonary TB from the GEO database to screen out 9272 differentially expressed mRNAs (*P* < 0.05). We screened out 16 potential downstream mRNAs for hsa-miR-409-5p, and their differential expression was analyzed using Jvenn. These mRNAs included RPS4X, NBEA, GSK3B, RGL2, ZNF512B, SOD2, ZNF12, KPNA3, AKAP1, RPRD2, ACAP2, RSU1, FDXR, EIF4EBP2, SRRD, and ZBTB34 (Figure 4(b)). Finally, the circRNA–miRNA–mRNA interaction network was constructed using Cytoscape (Figure 4(c)).

#### 3.4.2. Biological Function Analysis of the Identified Genes

**Figure 5:**
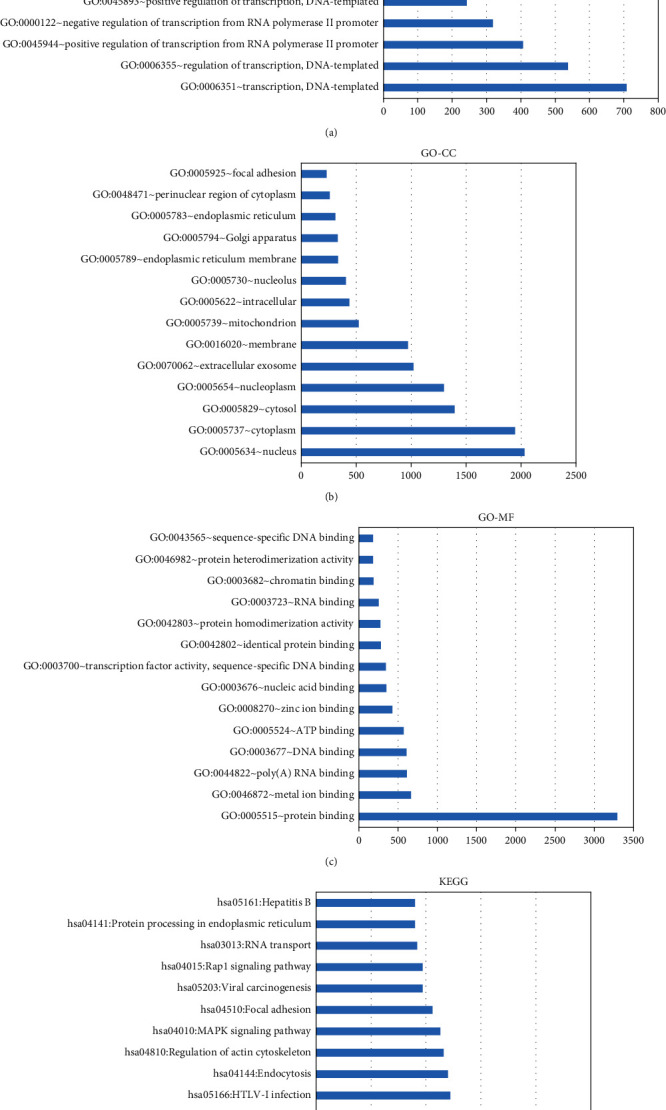
Biological function enrichment analysis. (a) Biological process (BP) enrichment analysis. (b) Cellular component (CC) enrichment analysis. (c) Molecular function (MF) enrichment analysis. (d) KEGG pathway enrichment analysis.

To study the effects of the identified mRNAs on pulmonary TB, we performed GO and KEGG analysis of the downstream mRNAs of the three hub circRNAs using the DAVID database. In BP analysis, DNA template, transcription, and positive regulation of transcription from RNA polymerase II promoter were the three terms with the highest enrichment (Figure 5(a)). Regarding CC, genes were enriched in the nucleus, cytoplasm, cytosol, nuclear plasma, and other components (Figure 5(b)). In MF analysis, protein binding, metal ion binding, and poly(A) RNA binding were the three terms with the highest enrichment (Figure 5(c)). Additionally, KEGG pathway analysis showed that the genes were enriched in the cancer pathway, PI3K-Akt signaling pathway, proteoglycan in cancer, Ras signaling pathway, HTLV-I infection, and other pathways (Figure 5(d)). Collectively, these results show that the downstream mRNAs of the three key circRNAs play a major immunomodulatory role in the development of pulmonary TB.

### 3.5. PPI Network Construction

**Figure 6:**
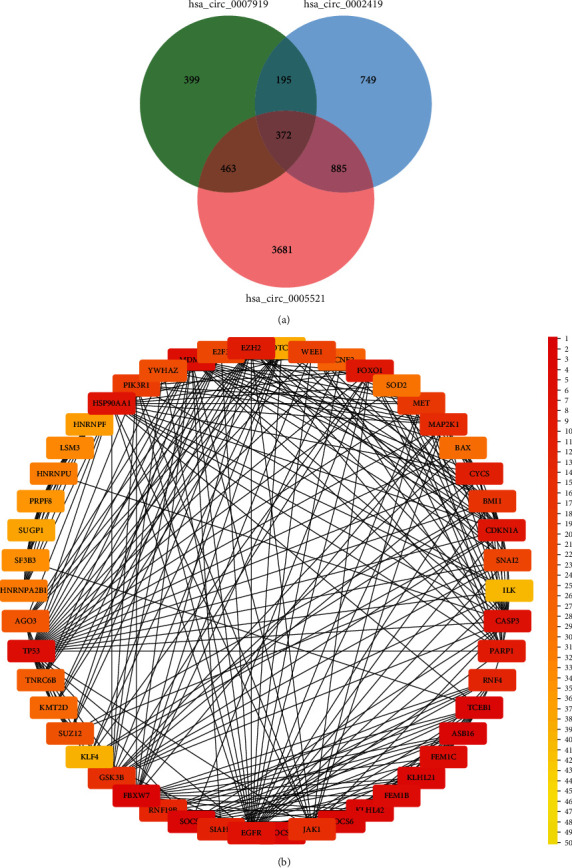
Screening the hub mRNAs. (a) Intersection analysis of potential downstream mRNAs of hsa_circ_0007919, hsa_circ_0002419, and hsa_circ_0005521. (b) PPI network of the top 50 hub mRNAs.

In this study, we have uncovered potential mRNAs downstream of three hub circRNAs. Among them, there were 1429 downstream mRNAs of hsa_circ_0007919, 2201 downstream mRNAs of hsa_circ_0002419, and 5401 downstream mRNAs of hsa_circ_0005521. We hypothesized that mRNAs simultaneously regulated by the three circRNAs may play important roles in the development of pulmonary TB. Therefore, we screened out 372 overlapping mRNAs using Jvenn and conducted further analysis (Figure 6(a)). To further find the hub genes among the 372 mRNAs in the network, we used the STRING database and Cytoscape. We found the top 50 hub genes according to the cytoHubba plugin in Cytoscape. We then constructed the PPI network on the basis of these top 50 genes (Table 6 and Figure 6(b)).

## 4. Discussion

Current laboratory tests for pulmonary TB include immunological- and molecular-based assays. Traditional smear staining has the disadvantages of a low positive rate and a long culture period [31]. With the further development of biomedical technologies, exosomal microRNA, real-time fluorescence quantitative PCR, and other techniques have been applied, but there is still a lack of rapid and reliable detection techniques for pulmonary TB [32, 33]. CircRNAs have several advantages as a biomarker, and their roles in the pathological regulation of pulmonary TB have received increasing attention in recent years [34]. Many studies have shown that circRNA expression is altered in the tissues and peripheral blood of pulmonary TB patients [35]. In this study, we uncovered three hub circRNAs by using differential gene expression, machine learning, and univariate analysis, including hsa_circ_0007919 (upregulated in diseased tissues) and hsa_circ_0002419 and hsa_circ_0005521 (downregulated in diseased tissues). To date, there have been no reports about hsa-circ-0002419 or hsa_circ_0005521. However, it has been found that potential downstream genes of hsa-miR-409-5p and hsa_circ_0005521 may also interact with hsa_circ_0028883, which has potential diagnostic value for pulmonary TB [36]. Wang et al. showed that hsa_circ_0007919 plays a role in ulcerative colitis by binding to hsa-miR-138 and hsa-let-7a to regulate the expression of VIPR1 and EPC1, respectively [37]. Pulmonary TB also includes chronic inflammation, and the two diseases may share common pathways in terms of inflammation.

We further studied the effects of potential genes downstream of the hub circRNAs on pulmonary TB. According to GO and KEGG analysis, the genes were enriched in the PI3K-Akt signaling pathway, the proteoglycan pathway in cancer, and Ras signaling. Yang et al. showed that the inflammatory response of macrophage-like cells to MTB can be attenuated by modulating the PI3K/Akt/mTOR signaling pathway [38]. Other studies have shown that PI3K/AKT/mTOR signaling pathways are suppressed in patients with active pulmonary TB [39]. Gill et al. explained that the mechanism by which proteoglycans modulate inflammatory responses in the lung and showed that they may be part of a new treatment for inflammatory lung diseases and lung infections [40]. It has also been shown that hsa_circRNA_103571, which is differently expressed in the plasma of patients with active pulmonary TB, is also involved in the Ras pathway [41].

We uncovered 50 hub genes and then constructed a PPI network using the STRING database and cytoHubba. FBXW7 scored the highest, and SOCS3 was the next highest. We concluded that these genes may be associated with the development of pulmonary TB. Additionally, some of these genes have been reported to be involved in the development of pulmonary TB and other diseases. FBXW7 is an important tumor suppressor. Ni et al. found that miR-92a plays an oncogene role in nonsmall cell lung cancer by regulating FBXW7 [42]. It has been reported that FBXW7 plays a key role in regulating colitis by inducing CCL2 and CCL7 expression in macrophages and promoting the accumulation of pro-inflammatory mononuclear macrophages [43]. Cui et al. found that inactivation of FBXW7 in cancer cells, especially those with wild-type p53, may improve the efficacy of radiotherapy or chemotherapy, and thus improve patient survival [44]. Pulmonary TB is a risk factor for lung cancer, and the probability of developing lung cancer is much higher in pulmonary TB patients than in the general population. Therefore, FBXW7 may play a role in pulmonary TB patients progressing to lung cancer through the above genes or pathways. SOCS3 is a suppressor of cytokine signaling; Feng et al. showed that stimulating G-protein-coupled receptor 120 (GPR120) induced SOCS3 expression and that GPR120-specific small molecule agonists improved autoimmune inflammation *via* dendritic cells [45]. Harling et al. showed that T cells are essential to prevent MTB infection and that T cell damage promotes the development of pulmonary TB. They concluded that high SOCS3 expression is a factor in the impaired T cell function of pulmonary TB patients [46].

However, this study had some limitations. First, expression levels of the DEGs need to be further verified by quantitative PCR. Second, the model with 100% prediction accuracy established in this study needs further parameter changes, optimization, and assessments before it can be widely used. Finally, the mechanisms of these DEGs in pulmonary TB need to be further explored by molecular experimentation.

## 5. Conclusions

In conclusion, we screened three hub circRNAs using the adjacent normal and diseased tissues of pulmonary TB patients. GO and KEGG enrichment were used to identify pathways associated with pulmonary TB. We identified hub genes through a PPI network. This study may provide a reference for finding candidate markers for the early diagnosis of pulmonary TB and provide new directions for possible pulmonary TB therapeutic targets.

## Figures and Tables

**Table 1:** The top 10 differentially expressed genes between normal and diseased tissues.

circBase_ID	ID	log2FC (H/L)	*P* value	Regulate
_	chr6:32489682|32549615	18.39	0.016744549	Up
_	chr6:31239376|31324219	15.76	5.20E-16	Up
_	chr6:29913011|29976954	15.27	6.44E-15	Up
_	chr12:103657104|103664086	11.4	0.007540201	Up
hsa_circ_0007919	chr17:953290|1003975	11.37	0.001677147	Up
hsa_circ_0049335	chr19:10906048|10909248	11.25	0.010820527	Up
_	chr5:94224584|94267696	3.67	0.021390567	Up
hsa_circ_0006272	chr10:70497602|70502326	3.45	0.026522304	Up
hsa_circ_0071410	chr4:169812073|169837178	3.41	0.012791713	Up
hsa_circ_0006006	chr2:173435454|173460751	3.36	0.022707405	Up
_	chr2:152109269|152112257	-11.18	0.045132825	Down
hsa_circ_0005281	chr17:80721841|80730383	-11.21	0.047352003	Down
_	chr11:36415396|36440853	-11.36	0.017056129	Down
_	chr8:108296910|108315595	-11.59	0.043394725	Down
hsa_circ_0002419	chr12:78443773|78452895	-11.69	0.001451403	Down
hsa_circ_0001961	chr10:52907842|52916908	-11.71	0.046807666	Down
hsa_circ_0037054	chr15:100185766|100215663	-11.9	0.030332661	Down
hsa_circ_0013225	chr1:94667276|94697199	-12.02	0.038719442	Down
hsa_circ_0057608	chr2:197777606|197786910	-12.05	0.024486018	Down
hsa_circ_0005521	chr1:215759838|215768813	-12.11	0.001277288	Down

**Table 2:** Feature screening information.

Feature-screening method	Number of circRNAs	Number of 100% correct algorithms
125 circRNA	125	3/13
CfsSubsetEval-BestFirst	14	3/13
PrincipalComponents-Ranker-T	5	0/13
CorrelationAttributeEval-Ranker-T	10	4/13
GainRatioAttributeEval-Ranker-T	12	0/13
InfoGainAttributeEval-Ranker-T	12	0/13
OneRAttributeEval-Ranker-T	16	2/13
ReliefFAttributeEval-Ranker-T	12	2/13
SymmetricalUncertAttributeEval-Ranker-T	12	0/13

**Table 3:** Frequency of occurrence of 29 circRNAs in four classes of feature-screened circRNAs.

Number	CircRNA	Number of occurrences
1	hsa_circ_0007919	4/4
2	chr10:15590454|15628663	4/4
3	hsa_circ_0002419	4/4
4	chr10:76729418|76748870	3/4
5	hsa_circ_0005521	3/4
6	chr12:97886239|97954825	3/4
7	chr10:97141442|97170534	2/4
8	hsa_circ_0034293	2/4
9	hsa_circ_0013048	2/4
10	hsa_circ_0007769	2/4
11	chr6:62362160|62442669	2/4
12	chrX:51070573|51099879	2/4
13	chr8:68934271|68973014	2/4
14	hsa_circ_0002286	2/4
15	hsa_circ_0006272	1/4
16	chr12:103657104|103664086	1/4
17	chr11:36415396|36440853	1/4
18	chr16:65005482|65026937	1/4
19	chr1:21231376|21268823	1/4
20	hsa_circ_0008336	1/4
21	hsa_circ_0008223	1/4
22	hsa_circ_0057105	1/4
23	chr7:115750762|115752092	1/4
24	hsa_circ_0066452	1/4
25	hsa_circ_0084708	1/4
26	hsa_circ_0080947	1/4
27	hsa_circ_0003961	1/4
28	hsa_circ_0042103	1/4
29	hsa_circ_0076948	1/4

**Table 4:** Univariate analysis of 14 circRNAs.

circRNA	SMO(%)	IBK(%)	HoeffdingTree(%)	Logistic(%)
hsa_circ_0007919	—	83	83	83
chr10:15590454|15628663	83	94	83	83
hsa_circ_0002419	94	94	94	94
chr10:76729418|76748870	83	—	83	83
hsa_circ_0005521	89	89	89	89
chr12:97886239|97954825	89	83	89	89
chr10:97141442|97170534	—	94	83	—
hsa_circ_0034293	—	83	89	—
hsa_circ_0013048	—	89	—	—
hsa_circ_0007769	83	89	83	—
chr6:62362160|62442669	—	83	83	83
chrX:51070573|51099879	83	—	—	—
chr8:68934271|68973014	83	83	—	—
hsa_circ_0002286	83	—	83	83

**Table 5:** Statistical analysis of downstream genes of the identified circRNAs.

circRNA	miRNA	mRNA
hsa_circ_0007919	13	1429
hsa_circ_0002419	15	2201
hsa_circ_0005521	46	5401

**Table 6:** Scores of the top 50 hub mRNAs.

mRNA	MCC
FBXW7	403816
SOCS3	403476
TCEB1	403213
ASB16	403206
KLHL21	403202
KLHL42	403200
SOCS6	362929
SOCS5	362928
FEM1B	362886
FEM1C	362882
TP53	139047
MDM2	135767
CASP3	124579
EGFR	123489
HSP90AA1	121780
CDKN1A	114332
FOXO1	94090
PARP1	92418
EZH2	59540
CYCS	57445
SIAH2	40341
RNF4	40323
RNF19B	40320
MAP2K1	22088
JAK1	16144
GSK3B	12798
BMI1	11425
MET	11221
PIK3R1	9141
WEE1	5886
SNAI2	5763
E2F3	3748
YWHAZ	2970
SUZ12	1957
AGO3	1848
TNRC6B	1825
CCNE2	1690
KMT2D	1119
BAX	864
SOD2	777
HNRNPA2B1	748
HNRNPU	736
LSM3	730
SF3B3	727
PRPF8	725
HNRNPF	724
SUGP1	720
NOTCH2	499
KLF4	442
ILK	265

## Data Availability

The data can be seen in PRJNA795290, GSE158767, GSE29190, and GSE83456 and the Supplementary Materials.

## Supplementary Materials

Supplementary Materials The 13 algorithms used to build the machine learning models are shown in Tables S1–S9 in the supplementary material.
