# Supplementary material for: Identification of Key CircRNAs Related to Pulmonary Tuberculosis Based on Bioinformatics Analysis
Source: Biomed Res Int. 2022 Apr 4;2022:1717784. doi: 10.1155/2022/1717784 (PMC9001091; doi:10.1155/2022/1717784)
Supplement: Supplementary Materials — The 13 algorithms used to build the machine learning models are shown in Tables S1–S9 in the supplementary material. [file 1717784.f1.docx]

Table S1: Model construction using 125 circRNAs

| Algorithm | Random seed(%) | | | | | | | | | | Average |
| --- | --- | --- | --- | --- | --- | --- | --- | --- | --- | --- | --- |
|  | 1 | 2 | 3 | 4 | 5 | 6 | 7 | 8 | 9 | 10 |  |
| ZeroR | 50 | 50 | 50 | 50 | 50 | 50 | 50 | 50 | 50 | 50 | 50 |
| Logistic | 100 | 100 | 100 | 100 | 100 | 100 | 100 | 100 | 100 | 100 | 100 |
| SMO | 100 | 100 | 100 | 100 | 100 | 100 | 100 | 100 | 100 | 100 | 100 |
| IBK | 100 | 100 | 100 | 100 | 100 | 100 | 100 | 100 | 100 | 100 | 100 |
| AttributeSelectedClassifier | 89 | 89 | 89 | 89 | 89 | 89 | 89 | 89 | 89 | 89 | 89 |
| ONER | 83 | 83 | 83 | 83 | 83 | 83 | 83 | 83 | 83 | 83 | 83 |
| DecisionStump | 67 | 67 | 67 | 67 | 67 | 67 | 67 | 67 | 67 | 67 | 67 |
| HoeffdingTree | 78 | 78 | 78 | 78 | 78 | 78 | 78 | 78 | 78 | 78 | 78 |
| J48 | 89 | 89 | 89 | 89 | 89 | 89 | 89 | 89 | 89 | 89 | 89 |
| LMT | 83 | 78 | 89 | 94 | 83 | 83 | 78 | 78 | 83 | 89 | 83.8 |
| RandomForest | 83 | 83 | 89 | 83 | 83 | 83 | 78 | 83 | 83 | 89 | 83.7 |
| RandomTree | 67 | 72 | 56 | 83 | 78 | 61 | 72 | 67 | 72 | 61 | 68.9 |
| REPTree | 50 | 44 | 61 | 33 | 50 | 50 | 22 | 44 | 39 | 61 | 45.4 |

Table S2: CfsSubsetEval-BestFirst

| Algorithm | Random seed(%) | | | | | | | | | | Average |
| --- | --- | --- | --- | --- | --- | --- | --- | --- | --- | --- | --- |
|  | 1 | 2 | 3 | 4 | 5 | 6 | 7 | 8 | 9 | 10 |  |
| ZeroR | 50 | 50 | 50 | 50 | 50 | 50 | 50 | 50 | 50 | 50 | 50 |
| Logistic | 94 | 94 | 94 | 94 | 94 | 94 | 94 | 94 | 94 | 94 | 94 |
| SMO | 100 | 100 | 100 | 100 | 100 | 100 | 100 | 100 | 100 | 100 | 100 |
| IBK | 100 | 100 | 100 | 100 | 100 | 100 | 100 | 100 | 100 | 100 | 100 |
| AttributeSelectedClassifier | 89 | 89 | 89 | 89 | 89 | 89 | 89 | 89 | 89 | 89 | 89 |
| ONER | 89 | 89 | 89 | 89 | 89 | 89 | 89 | 89 | 89 | 89 | 89 |
| DecisionStump | 83 | 83 | 83 | 83 | 83 | 83 | 83 | 83 | 83 | 83 | 83 |
| HoeffdingTree | 100 | 100 | 100 | 100 | 100 | 100 | 100 | 100 | 100 | 100 | 100 |
| J48 | 89 | 89 | 89 | 89 | 89 | 89 | 89 | 89 | 89 | 89 | 89 |
| LMT | 94 | 89 | 94 | 89 | 94 | 94 | 94 | 94 | 83 | 89 | 91.4 |
| RandomForest | 94 | 94 | 94 | 94 | 94 | 94 | 94 | 94 | 94 | 94 | 94 |
| RandomTree | 94 | 83 | 89 | 83 | 94 | 72 | 89 | 89 | 83 | 94 | 87 |
| REPTree | 72 | 72 | 83 | 67 | 72 | 72 | 56 | 61 | 61 | 83 | 69.9 |

Table S3: PrincipalComponents-Ranker-T

| Algorithm | Random seed(%) | | | | | | | | | | Average |
| --- | --- | --- | --- | --- | --- | --- | --- | --- | --- | --- | --- |
|  | 1 | 2 | 3 | 4 | 5 | 6 | 7 | 8 | 9 | 10 |  |
| ZeroR | 50 | 50 | 50 | 50 | 50 | 50 | 50 | 50 | 50 | 50 | 50 |
| Logistic | 67 | 67 | 67 | 67 | 67 | 67 | 67 | 67 | 67 | 67 | 67 |
| SMO | 78 | 78 | 78 | 78 | 78 | 78 | 78 | 78 | 78 | 78 | 78 |
| IBK | 72 | 72 | 72 | 72 | 72 | 72 | 72 | 72 | 72 | 72 | 72 |
| AttributeSelectedClassifier | 83 | 83 | 83 | 83 | 83 | 83 | 83 | 83 | 83 | 83 | 83 |
| ONER | 67 | 67 | 67 | 67 | 67 | 67 | 67 | 67 | 67 | 67 | 67 |
| DecisionStump | 83 | 83 | 83 | 83 | 83 | 83 | 83 | 83 | 83 | 83 | 83 |
| HoeffdingTree | 83 | 83 | 83 | 83 | 83 | 83 | 83 | 83 | 83 | 83 | 83 |
| J48 | 83 | 83 | 83 | 83 | 83 | 83 | 83 | 83 | 83 | 83 | 83 |
| LMT | 78 | 78 | 78 | 78 | 78 | 78 | 78 | 72 | 78 | 78 | 77.4 |
| RandomForest | 83 | 83 | 83 | 83 | 78 | 83 | 83 | 83 | 83 | 83 | 82.5 |
| RandomTree | 89 | 78 | 83 | 72 | 83 | 83 | 83 | 83 | 78 | 78 | 81 |
| REPTree | 67 | 67 | 61 | 72 | 72 | 56 | 72 | 83 | 72 | 72 | 69.4 |

Table S4: CorrelationAttributeEval-Ranker-T

| Algorithm | Random seed(%) | | | | | | | | | | Average |
| --- | --- | --- | --- | --- | --- | --- | --- | --- | --- | --- | --- |
|  | 1 | 2 | 3 | 4 | 5 | 6 | 7 | 8 | 9 | 10 |  |
| ZeroR | 50 | 50 | 50 | 50 | 50 | 50 | 50 | 50 | 50 | 50 | 50 |
| Logistic | 100 | 100 | 100 | 100 | 100 | 100 | 100 | 100 | 100 | 100 | 100 |
| SMO | 100 | 100 | 100 | 100 | 100 | 100 | 100 | 100 | 100 | 100 | 100 |
| IBK | 100 | 100 | 100 | 100 | 100 | 100 | 100 | 100 | 100 | 100 | 100 |
| AttributeSelectedClassifier | 89 | 89 | 89 | 89 | 89 | 89 | 89 | 89 | 89 | 89 | 89 |
| ONER | 89 | 89 | 89 | 89 | 89 | 89 | 89 | 89 | 89 | 89 | 89 |
| DecisionStump | 72 | 72 | 72 | 72 | 72 | 72 | 72 | 72 | 72 | 72 | 72 |
| HoeffdingTree | 100 | 100 | 100 | 100 | 100 | 100 | 100 | 100 | 100 | 100 | 100 |
| J48 | 89 | 89 | 89 | 89 | 89 | 89 | 89 | 89 | 89 | 89 | 89 |
| LMT | 94 | 94 | 94 | 94 | 94 | 94 | 94 | 94 | 94 | 94 | 94 |
| RandomForest | 89 | 94 | 83 | 89 | 89 | 89 | 83 | 89 | 83 | 89 | 87.7 |
| RandomTree | 72 | 89 | 72 | 89 | 83 | 89 | 83 | 94 | 83 | 72 | 82.6 |
| REPTree | 61 | 61 | 61 | 61 | 67 | 78 | 56 | 72 | 83 | 72 | 67.2 |

Table S5: GainRatioAttributeEval-Ranker-T

| Algorithm | Random seed(%) | | | | | | | | | | Average |
| --- | --- | --- | --- | --- | --- | --- | --- | --- | --- | --- | --- |
|  | 1 | 2 | 3 | 4 | 5 | 6 | 7 | 8 | 9 | 10 |  |
| ZeroR | 50 | 50 | 50 | 50 | 50 | 50 | 50 | 50 | 50 | 50 | 50 |
| Logistic | 94 | 94 | 94 | 94 | 94 | 94 | 94 | 94 | 94 | 94 | 94 |
| SMO | 83 | 83 | 83 | 83 | 83 | 83 | 83 | 83 | 83 | 83 | 83 |
| IBK | 83 | 83 | 83 | 83 | 83 | 83 | 83 | 83 | 83 | 83 | 83 |
| AttributeSelectedClassifier | 89 | 89 | 89 | 89 | 89 | 89 | 89 | 89 | 89 | 89 | 89 |
| ONER | 89 | 89 | 89 | 89 | 89 | 89 | 89 | 89 | 89 | 89 | 89 |
| DecisionStump | 83 | 83 | 83 | 83 | 83 | 83 | 83 | 83 | 83 | 83 | 83 |
| HoeffdingTree | 94 | 94 | 94 | 94 | 94 | 94 | 94 | 94 | 94 | 94 | 94 |
| J48 | 89 | 89 | 89 | 89 | 89 | 89 | 89 | 89 | 89 | 89 | 89 |
| LMT | 83 | 83 | 83 | 83 | 89 | 83 | 89 | 83 | 83 | 83 | 84.2 |
| RandomForest | 94 | 89 | 89 | 89 | 89 | 89 | 89 | 89 | 89 | 94 | 90 |
| RandomTree | 89 | 94 | 89 | 89 | 89 | 83 | 83 | 94 | 89 | 89 | 88.8 |
| REPTree | 78 | 78 | 89 | 89 | 78 | 78 | 61 | 78 | 78 | 83 | 79 |

Table S6: InfoGainAttributeEval-Ranker-T

| Algorithm | Random seed(%) | | | | | | | | | | Average |
| --- | --- | --- | --- | --- | --- | --- | --- | --- | --- | --- | --- |
|  | 1 | 2 | 3 | 4 | 5 | 6 | 7 | 8 | 9 | 10 |  |
| ZeroR | 50 | 50 | 50 | 50 | 50 | 50 | 50 | 50 | 50 | 50 | 50 |
| Logistic | 94 | 94 | 94 | 94 | 94 | 94 | 94 | 94 | 94 | 94 | 94 |
| SMO | 83 | 83 | 83 | 83 | 83 | 83 | 83 | 83 | 83 | 83 | 83 |
| IBK | 83 | 83 | 83 | 83 | 83 | 83 | 83 | 83 | 83 | 83 | 83 |
| AttributeSelectedClassifier | 89 | 89 | 89 | 89 | 89 | 89 | 89 | 89 | 89 | 89 | 89 |
| ONER | 89 | 89 | 89 | 89 | 89 | 89 | 89 | 89 | 89 | 89 | 89 |
| DecisionStump | 83 | 83 | 83 | 83 | 83 | 83 | 83 | 83 | 83 | 83 | 83 |
| HoeffdingTree | 94 | 94 | 94 | 94 | 94 | 94 | 94 | 94 | 94 | 94 | 94 |
| J48 | 89 | 89 | 89 | 89 | 89 | 89 | 89 | 89 | 89 | 89 | 89 |
| LMT | 83 | 83 | 83 | 83 | 89 | 83 | 89 | 83 | 83 | 83 | 84.2 |
| RandomForest | 94 | 89 | 89 | 89 | 89 | 89 | 89 | 89 | 89 | 94 | 90 |
| RandomTree | 83 | 67 | 83 | 83 | 78 | 83 | 89 | 89 | 89 | 83 | 82.7 |
| REPTree | 78 | 67 | 89 | 78 | 78 | 72 | 67 | 78 | 89 | 83 | 77.9 |

Table S7: OneRAttributeEval-Ranker-T

| Algorithm | Random seed(%) | | | | | | | | | | Average |
| --- | --- | --- | --- | --- | --- | --- | --- | --- | --- | --- | --- |
|  | 1 | 2 | 3 | 4 | 5 | 6 | 7 | 8 | 9 | 10 |  |
| ZeroR | 50 | 50 | 50 | 50 | 50 | 50 | 50 | 50 | 50 | 50 | 50 |
| Logistic | 94 | 94 | 94 | 94 | 94 | 94 | 94 | 94 | 94 | 94 | 94 |
| SMO | 100 | 100 | 100 | 100 | 100 | 100 | 100 | 100 | 100 | 100 | 100 |
| IBK | 100 | 100 | 100 | 100 | 100 | 100 | 100 | 100 | 100 | 100 | 100 |
| AttributeSelectedClassifier | 89 | 89 | 89 | 89 | 89 | 89 | 89 | 89 | 89 | 89 | 89 |
| ONER | 89 | 89 | 89 | 89 | 89 | 89 | 89 | 89 | 89 | 89 | 89 |
| DecisionStump | 83 | 83 | 83 | 83 | 83 | 83 | 83 | 83 | 83 | 83 | 83 |
| HoeffdingTree | 83 | 83 | 83 | 83 | 83 | 83 | 83 | 83 | 83 | 83 | 83 |
| J48 | 89 | 89 | 89 | 89 | 89 | 89 | 89 | 89 | 89 | 89 | 89 |
| LMT | 89 | 94 | 89 | 94 | 89 | 94 | 89 | 94 | 89 | 94 | 91.5 |
| RandomForest | 83 | 89 | 83 | 83 | 83 | 83 | 83 | 83 | 89 | 89 | 84.8 |
| RandomTree | 83 | 61 | 83 | 72 | 83 | 89 | 100 | 83 | 83 | 89 | 82.6 |
| REPTree | 67 | 72 | 78 | 89 | 72 | 83 | 78 | 83 | 94 | 83 | 79.9 |

Table S8: CfsSubsetEval-BestFirst

| Algorithm | Random seed(%) | | | | | | | | | | Average |
| --- | --- | --- | --- | --- | --- | --- | --- | --- | --- | --- | --- |
|  | 1 | 2 | 3 | 4 | 5 | 6 | 7 | 8 | 9 | 10 |  |
| ZeroR | 50 | 50 | 50 | 50 | 50 | 50 | 50 | 50 | 50 | 50 | 50 |
| Logistic | 100 | 100 | 100 | 100 | 100 | 100 | 100 | 100 | 100 | 100 | 100 |
| SMO | 100 | 100 | 100 | 100 | 100 | 100 | 100 | 100 | 100 | 100 | 100 |
| IBK | 94 | 94 | 94 | 94 | 94 | 94 | 94 | 94 | 94 | 94 | 94 |
| AttributeSelectedClassifier | 89 | 89 | 89 | 89 | 89 | 89 | 89 | 89 | 89 | 89 | 89 |
| ONER | 89 | 89 | 89 | 89 | 89 | 89 | 89 | 89 | 89 | 89 | 89 |
| DecisionStump | 72 | 72 | 72 | 72 | 72 | 72 | 72 | 72 | 72 | 72 | 72 |
| HoeffdingTree | 89 | 89 | 89 | 89 | 89 | 89 | 89 | 89 | 89 | 89 | 89 |
| J48 | 89 | 89 | 89 | 89 | 89 | 89 | 89 | 89 | 89 | 89 | 89 |
| LMT | 94 | 94 | 94 | 94 | 94 | 94 | 94 | 94 | 94 | 94 | 94 |
| RandomForest | 89 | 83 | 89 | 89 | 83 | 89 | 78 | 83 | 89 | 89 | 86.1 |
| RandomTree | 67 | 83 | 67 | 56 | 72 | 72 | 83 | 72 | 72 | 89 | 73.3 |
| REPTree | 61 | 61 | 61 | 67 | 61 | 78 | 61 | 72 | 83 | 72 | 67.7 |

Table S9: ReliefFAttributeEval-Ranker-T

| Algorithm | Random seed(%) | | | | | | | | | | Average |
| --- | --- | --- | --- | --- | --- | --- | --- | --- | --- | --- | --- |
|  | 1 | 2 | 3 | 4 | 5 | 6 | 7 | 8 | 9 | 10 |  |
| ZeroR | 50 | 50 | 50 | 50 | 50 | 50 | 50 | 50 | 50 | 50 | 50 |
| Logistic | 94 | 94 | 94 | 94 | 94 | 94 | 94 | 94 | 94 | 94 | 94 |
| SMO | 83 | 83 | 83 | 83 | 83 | 83 | 83 | 83 | 83 | 83 | 83 |
| IBK | 83 | 83 | 83 | 83 | 83 | 83 | 83 | 83 | 83 | 83 | 83 |
| AttributeSelectedClassifier | 89 | 89 | 89 | 89 | 89 | 89 | 89 | 89 | 89 | 89 | 89 |
| ONER | 89 | 89 | 89 | 89 | 89 | 89 | 89 | 89 | 89 | 89 | 89 |
| DecisionStump | 83 | 83 | 83 | 83 | 83 | 83 | 83 | 83 | 83 | 83 | 83 |
| HoeffdingTree | 94 | 94 | 94 | 94 | 94 | 94 | 94 | 94 | 94 | 94 | 94 |
| J48 | 89 | 89 | 89 | 89 | 89 | 89 | 89 | 89 | 89 | 89 | 89 |
| LMT | 83 | 83 | 83 | 83 | 89 | 83 | 89 | 83 | 83 | 83 | 84.2 |
| RandomForest | 89 | 83 | 94 | 89 | 89 | 89 | 89 | 89 | 89 | 94 | 89.4 |
| RandomTree | 78 | 89 | 72 | 89 | 89 | 78 | 83 | 72 | 83 | 78 | 81.1 |
| REPTree | 78 | 78 | 89 | 89 | 78 | 78 | 61 | 78 | 78 | 83 | 79 |
